# The long distance transport of airborne *Ambrosia* pollen to the UK and the Netherlands from Central and south Europe

**DOI:** 10.1007/s00484-016-1170-7

**Published:** 2016-04-27

**Authors:** Letty A. de Weger, Catherine H. Pashley, Branko Šikoparija, Carsten A. Skjøth, Idalia Kasprzyk, Łukasz Grewling, Michel Thibaudon, Donat Magyar, Matt Smith

**Affiliations:** 1 Department of Pulmonology, Leiden University Medical Centre, PO Box 9600, 2300RC Leiden, The Netherlands; 2 Institute for Lung Health, Department of Infection, Immunity and Inflammation, University of Leicester, Leicester, UK; 3 Laboratory for Palynology, Department of Biology and Ecology, Faculty of Sciences, University of Novi Sad, Novi Sad, Serbia; 4 BioSense Institute, Research Institute for Information Technologies in Biosystems, University of Novi Sad, Novi Sad, Serbia; 5 National Pollen and Aerobiology Research Unit, Institute of Science and the Environment, University of Worcester, Henwick Grove, Worcester, UK; 6 Department of Environmental Biology, University of Rzeszów, Rzeszów, Poland; 7 Laboratory of Aeropalynology, Faculty of Biology, Adam Mickiewicz University, Poznań, Poland; 8 Reseau National de Surveillance Aerobiologique (RNSA), Brussieu, France; 9 Department of Aerobiology and Air Hygiene, National Public Health Center, Budapest, Hungary

**Keywords:** *Ambrosia*, Long distance transport, Back trajectory analysis, Atmospheric movement, Pannonian Plain

## Abstract

The invasive alien species *Ambrosia artemisiifolia* (common or short ragweed) is increasing its range in Europe. In the UK and the Netherlands, airborne concentrations of *Ambrosia* pollen are usually low. However, more than 30 *Ambrosia* pollen grains per cubic metre of air (above the level capable to trigger allergic symptoms) were recorded in Leicester (UK) and Leiden (NL) on 4 and 5 September 2014. The aims of this study were to determine whether the highly allergenic *Ambrosia* pollen recorded during the episode could be the result of long distance transport, to identify the potential sources of these pollen grains and to describe the conditions that facilitated this possible long distance transport. Airborne *Ambrosia* pollen data were collected at 10 sites in Europe. Back trajectory and atmospheric dispersion calculations were performed using HYSPLIT_4. Back trajectories calculated at Leicester and Leiden show that higher altitude air masses (1500 m) originated from source areas on the Pannonian Plain and Ukraine. During the episode, air masses veered to the west and passed over the Rhône Valley. Dispersion calculations showed that the atmospheric conditions were suitable for *Ambrosia* pollen released from the Pannonian Plain and the Rhône Valley to reach the higher levels and enter the airstream moving to northwest Europe where they were deposited at ground level and recorded by monitoring sites. The study indicates that the *Ambrosia* pollen grains recorded during the episode in Leicester and Leiden were probably not produced by local sources but transported long distances from potential source regions in east Europe, i.e. the Pannonian Plain and Ukraine, as well as the Rhône Valley in France.

**Electronic supplementary material:**

The online version of this article (doi:10.1007/s00484-016-1170-7) contains supplementary material, which is available to authorized users.

## Introduction

Allergic sensitization can result in disorders of the airways such as allergic rhinitis, conjunctivitis and allergic asthma (Zheng et al. 2011). Pollen grains from wind-pollinating (anemophilous) plants are often the causative agents of sensitization (Bousquet et al. 2007). Pollen from *Ambrosia* plants is one of the most relevant allergens in the USA (Oswalt and Marshall 2008) and is becoming an increasing problem in Europe. *Ambrosia* was accidentally introduced into Europe at the end of the nineteenth century. Since then, the plant has been steadily conquering Europe causing harm to agriculture and to public health (Smith et al. 2013). The most infested areas of Europe are currently the Rhône Valley in France, northern Italy, the Pannonian Plain, and large areas in Ukraine and western Russia (Skjøth et al. 2010; Smith et al. 2013; Thibaudon et al. 2014; Prank et al. 2013; Podberezko et al. 2013; Reznik 2009). Concomitantly, with the increase in plant abundance, there has been an increase in the number of patients sensitized to *Ambrosia*: ~60 % in Hungary (Makra et al. 2004); ~ 47 % in France, mainly the Rhône Valley (Thibaudon et al. 2010); and an increase from 24 % in 1989 to 70 % in 2008 was witnessed in northern Italy (Tosi et al. 2011). In countries like Spain and the UK, the *Ambrosia* sensitization rate is still low (Bousquet et al. 2007), corresponding with the scarcity of the plant in these areas.

*Ambrosia* seeds are constantly being introduced into Europe via imported grain and animal fodder resulting in areas around entry points, such as harbours or airports, being heavily infested by *Ambrosia*. Recent studies suggest a progress of the plant into Germany (e.g. Berlin, (Starfinger 2008)) and to a lesser extent the Netherlands (de Weger et al. 2009; Smith et al. 2013). In the Netherlands, most of the observations are of single plants or very small populations, often in private gardens, and probably originating from bird seed. However, recent analysis showed that there has been a small increase in the number of larger populations (>50 plants) in public spaces (Beringen et al. 2014; Smith et al. 2013). In the UK, *Ambrosia* is primarily an alien invasive plant of open, ruderal habitats (Essl et al. 2015). *Ambrosia* plants require long-lasting autumns and a late first-frost for their seeds to mature, which limits their northward distribution in Europe. Recent studies based upon climate change prediction models have suggested that habitat suitable for *Ambrosia* range expansion will extend further north and east such that it will become established in Scandinavian countries and Britain by 2050 (Hamaoui-Laguel et al. 2015; Storkey et al. 2014).

In regions that scarcely record any *Ambrosia* pollen, occasional peaks in atmospheric *Ambrosia* pollen concentrations are likely to be caused by long distance transport (LDT) from sources hundreds of kilometres away (e.g. (Belmonte et al. 2000; Fernández-Llamazares et al. 2012; Makra et al. 2010; Cecchi et al. 2006; Smith et al. 2013)). Studies in Poland using back trajectory analysis showed that peaks in airborne *Ambrosia* pollen recorded during the night and early in the morning were most likely brought by air masses loaded with pollen from the southern areas, like the Czech Republic, Slovakia and Hungary (Smith et al. 2008; Stach et al. 2007). Similarly, Kasprzyk et al. (2011) showed that Ukraine may be a source area of *Ambrosia* pollen for Poland.

Airborne concentrations of *Ambrosia* pollen are usually low in the UK and the Netherlands, generally not exceeding 10 pollen grains per year (de Weger et al. 2009; Pashley et al. 2015). The climatic conditions in these countries are not currently favourable for fulfilling the full life cycle of *Ambrosia*. The late flowering of the plant combined with the early dates of the first frosts in autumn prevents the *Ambrosia* seeds from ripening. However, future climate scenarios for the Netherlands (Klein Tank et al., 2014) and for Europe (Storkey et al. 2014) have suggested that *Ambrosia* could spread and persistent as far north as central England by the year 2050, with areas where *Ambrosia* populations are currently classed as casual becoming established. It is important to prevent the plant from becoming established in new regions since examples from other European countries have shown the dramatic increase in *Ambrosia* sensitization once this occurs. It is therefore imperative to routinely monitor for airborne *Ambrosia* pollen as this can be an early warning of invasion by the plant. Such routine monitoring revealed that, at the beginning of September 2014, more than 30 *Ambrosia* pollen grains per cubic metre of air were recorded in Leicester (UK) and Leiden (Netherlands), where there are no known local stands of *Ambrosia* plants. The aims of this study were to (1) determine whether this episode could be the result of LDT, since local sources are not known to be present; (2) identify the potential sources of these pollen grains; (3) try to describe the conditions that facilitated this possible episode of LDT that resulted in unusually high atmospheric concentrations of *Ambrosia* pollen.

## Materials and methods

### Pollen data

**Fig. 1:**
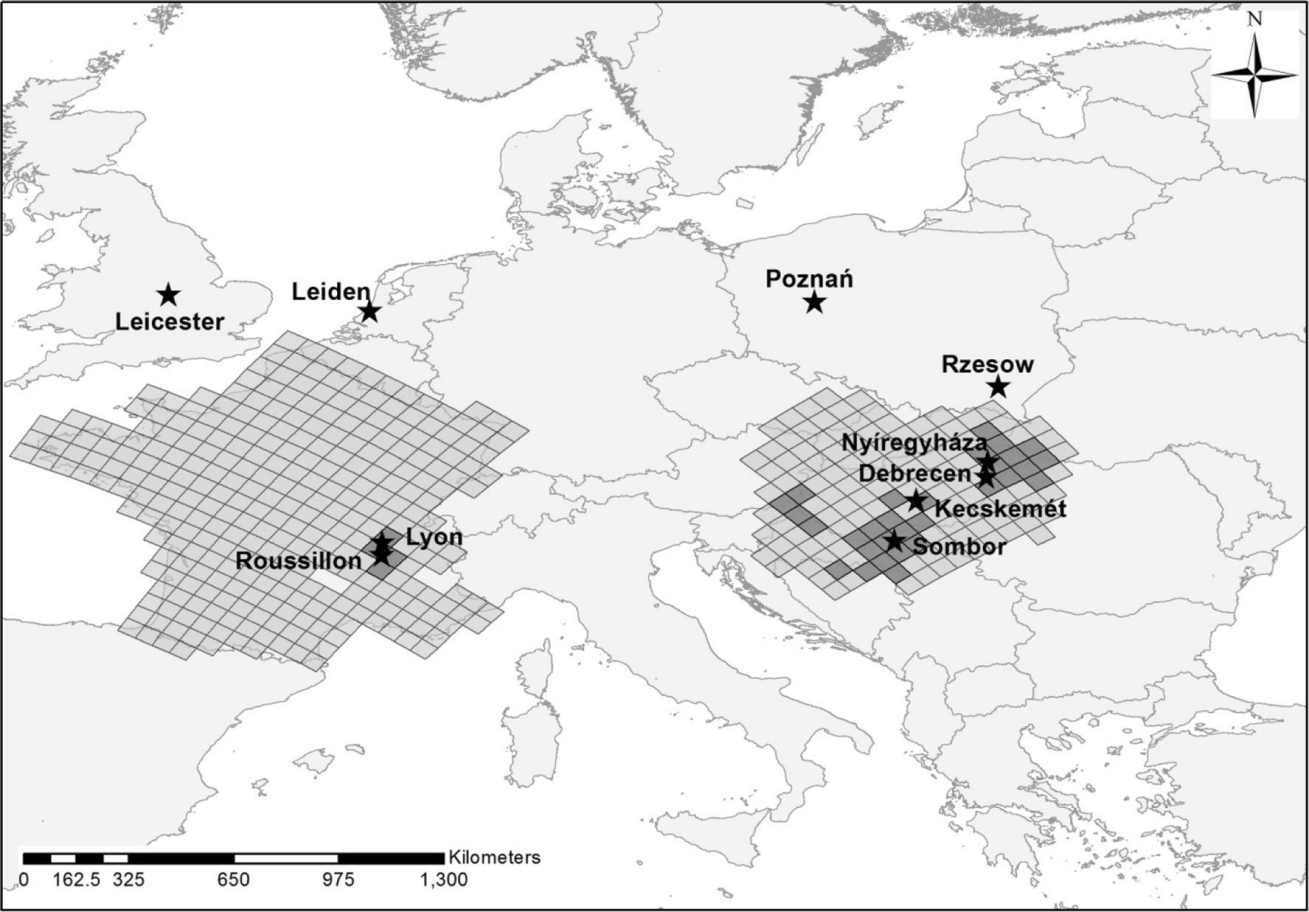
Distribution of the aerobiological monitoring stations used in this study and *Ambrosia* pollen source inventories. *Dark grey* indicates grid cells entered into the dispersion model, corresponding to the areas with the highest *Ambrosia* plant infestation according to the inventories by Skjøth et al. (2010) and Thibaudon et al. (2014)

*Ambrosia* pollen data were collected at 10 sites in Europe (Fig. 1) by volumetric spore traps of the Hirst design (Hirst 1952). Daily average and bi-hourly *Ambrosia* pollen concentrations are expressed as pollen grains per cubic metre of air (P m^−3^).

### Meteorological data

The overall synoptic weather situation was investigated using analysed weather maps from the UK Met Office, as well as reanalysed meteorological data and meteorological observations obtained from the National Centre for Environmental Prediction (NCEP) using the methodology given by Stach et al. (2007) and Kasprzyk et al. (2011). Synoptic charts were obtained from the website: http://www.wetterzentrale.de/topkarten/tkfaxbraar.htm.

### Back trajectory analysis

Back trajectory calculations were conducted using the HYSPLIT_4 (HYbrid Single-Particle Lagrangian Integrated Trajectory) model (Draxler et al. 2009). 3D back trajectories were calculated 72 h back in time, at five heights above ground level (200, 500, 1000, 1500 and 2000 m), for bi-hourly periods corresponding to pollen records in Leicester and Leiden on 4 and 5 September 2014. Trajectory calculations involve an amount of uncertainty, and this uncertainty increases exponentially with time. This is a drawback of using individual back trajectories (Stohl and Seibert 1998). Therefore, to account for this uncertainty, clusters based on nine trajectories with receptor points placed 0.5° apart were calculated. Trajectories of the cluster will be closely related until the trajectories reach a certain area, where even small variations in meteorology will create large variations in the transport path of the individual trajectories. All calculated trajectories examined in this study showed little variation with respect to transport path (Stach et al. 2007).

Input meteorological data for 1–7 September 2014 came from the Global Data Analysis System (GDAS) dataset provided by the NCEP that covers the period 2006 to present in the form of a 1° latitude-longitude grid (https://ready.arl.noaa.gov/gdas1.php).

### Dispersion modelling

Particle dispersion calculations were carried out with the HYSPLIT_4 model in order to determine whether atmospheric conditions during the studied episode would have allowed *Ambrosia* pollen to reach high altitude air masses after release in the source areas and to settle down in Leiden and Leicester following atmospheric transport.

In order to verify whether airborne *Ambrosia* pollen released in the source area could reach the altitudinal range of back trajectories arriving at Leiden, the model was set to release 2500 particles of 20 μm at 15 m above the ground each hour from 6 to 12 h, which corresponds to the most intensive period of *Ambrosia* pollen release (Barnes et al. 2001; Martin et al. 2010). Sedimentation processes are accounted for in the model by setting the settling velocity of the particles to 0.0156 m/s which corresponds to the settling velocity of *Ambrosia artemisiifolia* pollen grains (Raynor et al. 1970) and applying the conversion module that deposits each particle rather than reduce their mass.

The starting locations for the particles released into the dispersion model were previously identified as being the most important source areas for *Ambrosia* pollen on the Pannonian Plain (Skjøth et al. 2010) and France (Thibaudon et al. 2014) (Fig. 1). These source areas had been identified by the use of detailed knowledge of *Ambrosia* ecology, land cover information and spatial variations in the annual sum of atmospheric *Ambrosia* pollen concentrations.

Simulations of particle deposition using the HYSPLIT_4 were conducted again in order to determine whether *Ambrosia* pollen travelling at the height of air masses (as described by back trajectories) could settle out from the atmosphere to reach ground level monitoring sites. The dispersion model was set to run so that the released particles arrived at Leicester and Leiden at 12–14 h on 5 September, which was the time when the highest *Ambrosia* pollen concentrations were recorded (Table 1). The emission points were selected based on the results of trajectory analysis (9 points for each trajectory in the cluster). Particles were released at an altitude of ~1500 m in the path of the air masses travelling to Leicester (Suppl. Table 1) and Leiden (Suppl. Table 2). The model was set to release 500 particles per hour for 8 h (until the end of the period when the highest bi-hourly *Ambrosia* pollen concentrations were recorded). The total amount of released particles corresponds to approximately 20 % of the pollen (19,316 P m^−3^) that reached the altitudinal range of air masses that passed over the Pannonian Plain on the way to Leiden.

**Table 1:** The height of air masses arriving at Leiden after passing through the areas (Pannonian Plain or Rhône Valley) where the particle clouds were dispersed, and percentage of particles calculated to be present at each trajectory height range

	Pannonian Plain		Rhône Valley	
Time at which trajectories arrived in Leiden	Trajectory height (m)	% of particles dispersed in trajectory height	Trajectory height (m)	% of particles dispersed in trajectory height
4th September 02:00^a^	–	–	–	–
4th September 04:00	1233.9–1965.0	15.3	–	–
4th September 06:00	1211.6–1744.2	13.4	–	–
4th September 08:00	1097.9–1762.5	18.1	–	–
4th September 10:00	1332.1–1975.3	12.2	–	–
4th September 12:00	1164.3–2236.9	19.7	–	–
4th September 14:00	1114.9–2295.2	22.0	–	–
4th September 16:00	1018.3–2391.5	26.4	–	–
4th September 18:00	1112.0–2190.2	21.6	–	–
4th September 20:00	1190.9–1924.7	16.4	–	–
4th September 22:00	1212.9–1945.8	15.9	–	–
5th September 00:00	889.0–1834.3	28.7	–	–
5th September 02:00	785.6–2110.6	37.2	–	–
5th September 04:00	744.6–2300.4	40.5	–	–
5th September 06:00	685.0–2322.7	44.3	–	–
5th September 08:00	519.7–2234.8	55.6	–	–
5th September 10:00	454.9–2348.1	60.6	–	–
5th September 12:00	316.3–2234.5	71.0	1565.9–1648.0	1.1
5th September 14:00	337.5–3624.7	70.4	1573.0–2482.1	2.3
5th September 16:00	495.9–3488.8	58.3	1471.8–2237.2	4.9
5th September 18:00	593.7–3570.2	51.5	1554.2–2316.7	2.6
5th September 20:00	689.2–2981.8	44.8	1583.2–2438.2	2.1
5th September 22:00	591.8–2184.1	50.3	1605.1–2346.4	1.8
Average	854.2–2348.5	36.1	1558.9–2244.8	2.5

^a^Trajectory did not pass over the areas where the particles were dispersed

## Results and discussion

Unusually high daily average concentrations of airborne *Ambrosia* pollen, in excess of 30 P m^−3^, were recorded in Leicester (4–5 Sept 2014) and Leiden (3–5 Sept 2014) (Suppl. Figure 1 and Suppl. Table 3). Bi-hourly concentrations of *Ambrosia* pollen began to peak during the night and early morning and continued into the following day in both cities. These diurnal patterns suggested that the pollen grains did not originate from local sources, since studies have shown that *Ambrosia* pollen from local plants is usually recorded in the air from about 6.30 am to around midday (Ogden et al. 1969). Furthermore, the geographical scope of the episode, recorded in both Leicester and Leiden, suggests that this was not a localized phenomenon caused by emission from local populations (Sommer et al. 2015).

**Fig. 2:**
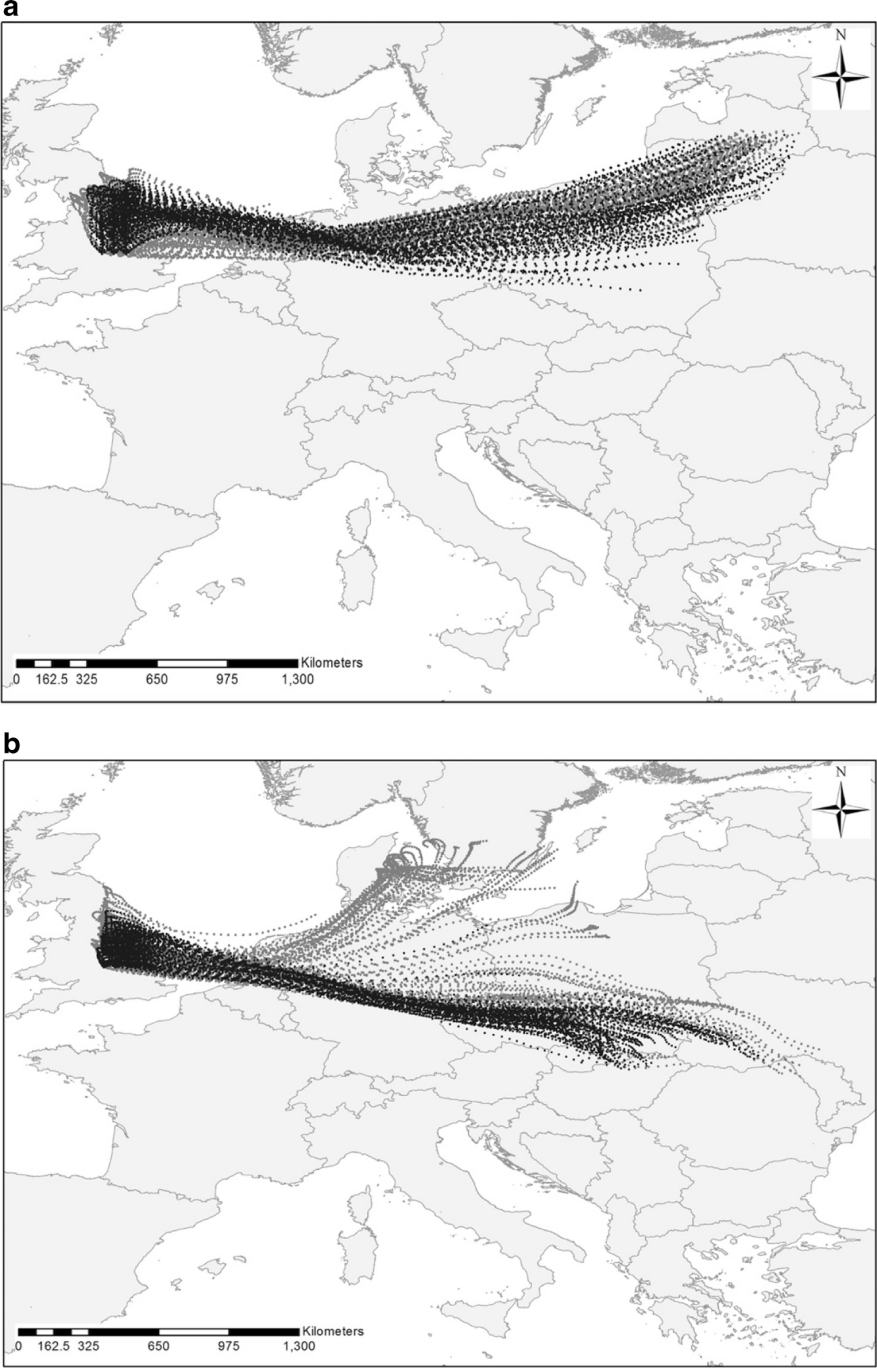
Clusters of 72-h backward trajectories calculated every 2 h 4th–5th September from Leicester at 500 (**a**) and 1500 m (**b**). The *light grey colour* indicates trajectories arriving when *Ambrosia* pollen was not recorded

**Fig. 3:**
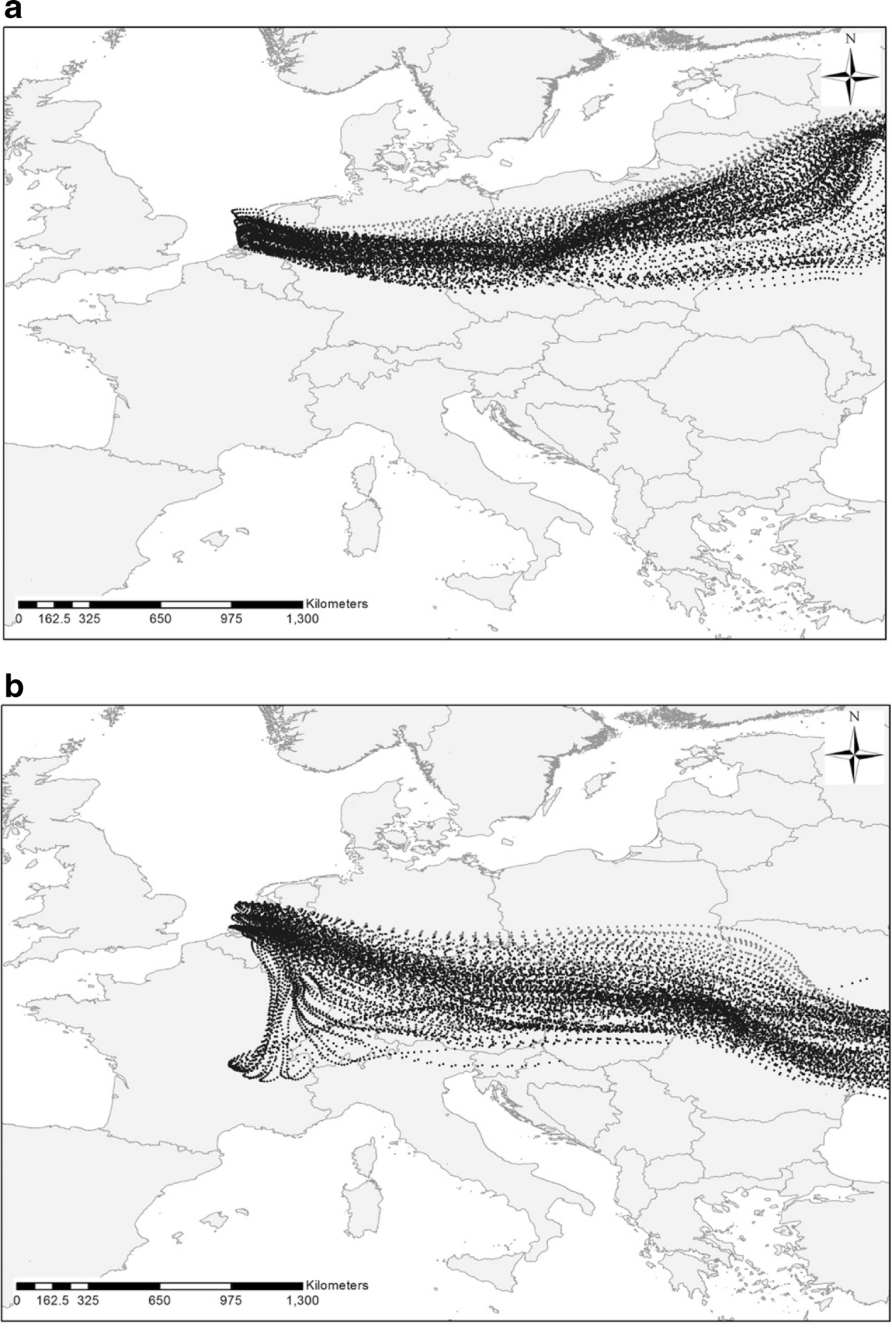
Clusters of 72-h backward trajectories calculated every 2 h 4th–5th September from Leiden at 500 (**a**) and 1500 m (**b**). The *light grey colour* indicates trajectories arriving when *Ambrosia* pollen was not recorded

Back trajectory analyses show that air masses arriving at Leicester (Fig. 2) and Leiden (Fig. 3) on the 4 and 5 September came from an easterly direction. The analyses were performed for various altitudes, but only those air masses arriving at Leicester and Leiden at higher altitudes (e.g. 1500 m above ground level (AGL)) passed over potential source areas on the Pannonian Plain (Skjøth et al. 2010) and Ukraine (Kasprzyk et al. 2011). Lower altitude air masses (e.g. 500 m AGL) tended to arrive from more northerly regions. It is interesting to note that the back trajectories calculated from Leicester mainly pass over Ukraine, rather than the Pannonian Plain. Whereas, the higher altitude air masses arriving at Leiden spent a considerable amount of time over the Pannonian Plain. However, the air masses arriving at Leicester passed close to Leiden where it is likely that mixing took place, indicating that both the Pannonian Plain and Ukraine were potential sources of airborne *Ambrosia* pollen at the two sites.

The idea that the *Ambrosia* pollen grains recorded in Leicester and Leiden were transported by high altitude air masses is supported by the fact that bi-hourly concentrations of *Ambrosia* pollen up to 377 P m^−3^ were recorded on the 2–3 September at Rzeszów, in Southeast Poland, which is located along the path taken by the high-level air masses travelling from Ukraine. On the other hand, very little airborne *Ambrosia* pollen (bi-hourly concentrations ≤5 P m^−3^) was recorded at this time in Poznań, in Western Poland, which lies on the path taken by the lower altitude air masses that approached from more northerly regions where notable sources of *Ambrosia* pollen have not been recorded (Suppl. Table 3, Figs. 2 and 3).

During the period 3–5 Sept. 2014, the synoptic situation was dominated by low-pressure systems (993–1012 hPa) residing over the Atlantic to the north of the British Isles and a high-pressure system (1029–1031 hPa) situated over the Baltic and European Russia. An occlusion was positioned over Poland, Denmark and Germany, particularly during the 1–4 September. This occluded front generally ran from east to west and marked the route taken by the pollen. It also helped to direct the warm air masses from Ukraine and the Pannonian Plain up in to the atmosphere. The result was that several different air masses lay on top of one another (the definition of an occlusion) and caused the lower parts of the atmosphere to have a different origin compared to the upper part.

**Fig. 4:**
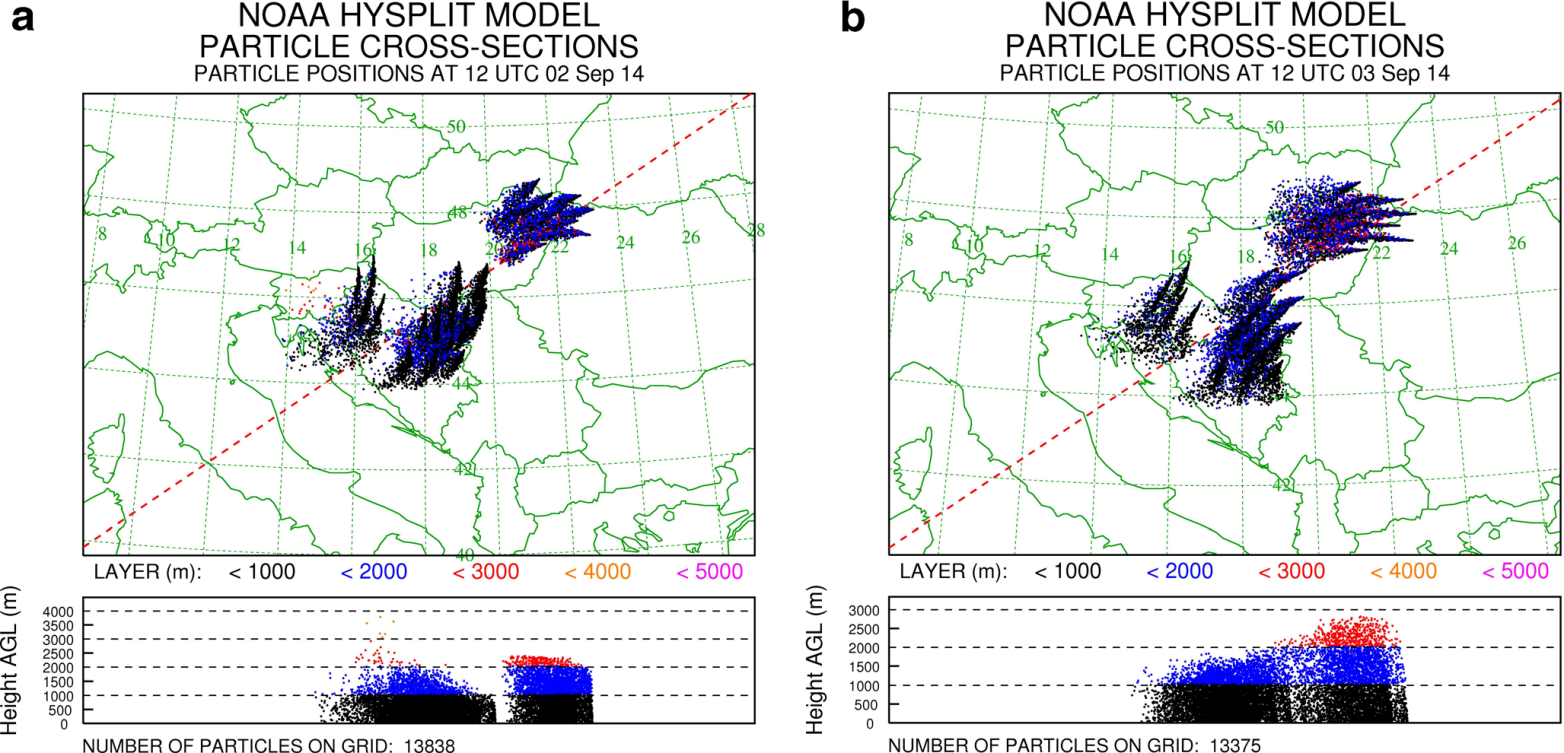
The output of the HYSPLIT model calculations of the distribution of particles released from 6 to 12 am at the Pannonian Plain on 2 September (**a**) and 3 September (**b**)

Pollen monitoring stations on the Pannonian Plain, i.e. Kecskemét, Debrecen, Nyíregyháza and Sombor, recorded bi-hourly concentrations of *Ambrosia* pollen in the range of 1000 to 4000 P m^−3^ during 1–6 September (Suppl. Table 3). It is likely that these pollen levels were of sufficient magnitude to allow large amounts of airborne *Ambrosia* pollen grains to be transported long distances (Šikoparija et al. 2013; Smith et al. 2008). In order to test the hypothesis that the Pannonian Plain could be a source of the *Ambrosia* pollen recorded in Leicester and Leiden, the HYSPLIT_4 dispersion model was run to determine whether the locally produced *Ambrosia* pollen could reach high enough altitudes to become entrained in high-level air flows moving towards northwest Europe. The calculations were made using the *Ambrosia* pollen source inventory produced by Skjøth et al. (2010) (Fig. 1). After release from heavily infested source areas on the Pannonian Plain on 2–3 September (6–12 h), an average of 36.1 % of the particles remaining airborne reached between 316.3 and 3624.7 m, which is the altitudinal range of back trajectories arriving at Leiden at the same time when the *Ambrosia* pollen was recorded (Table 1). Interestingly, only *Ambrosia* pollen grains released from sources in northern parts of the Pannonian Plain travelled northward and were able to enter the airstream travelling towards northwest Europe. Dispersion from sources located on southern parts of the Pannonian Plain tended to go south (Fig. 4). Unfortunately, a detailed inventory for *Ambrosia* pollen sources, as described for the Pannonian Plain (Skjøth et al. 2010) and France (Thibaudon et al. 2014), does not exist for Ukraine and so the analysis could not be repeated for this area.

Further investigation showed that towards the end of the episode, air masses calculated for 1500 m, which arrived at Leiden between 12 and 22 h on 5 September 2014, veered south and approached from the direction of potential source regions in the Rhône Valley in France (Fig. 3b). At the pollen monitoring station of Roussillon, bi-hourly concentrations of airborne *Ambrosia* pollen between 642 and 1085 P m^−3^ were recorded during the morning of 4 September (Suppl. Table 3), which is the time period that the air masses dwelled around in the Rhône Valley before moving to Leiden.

**Fig. 5:**
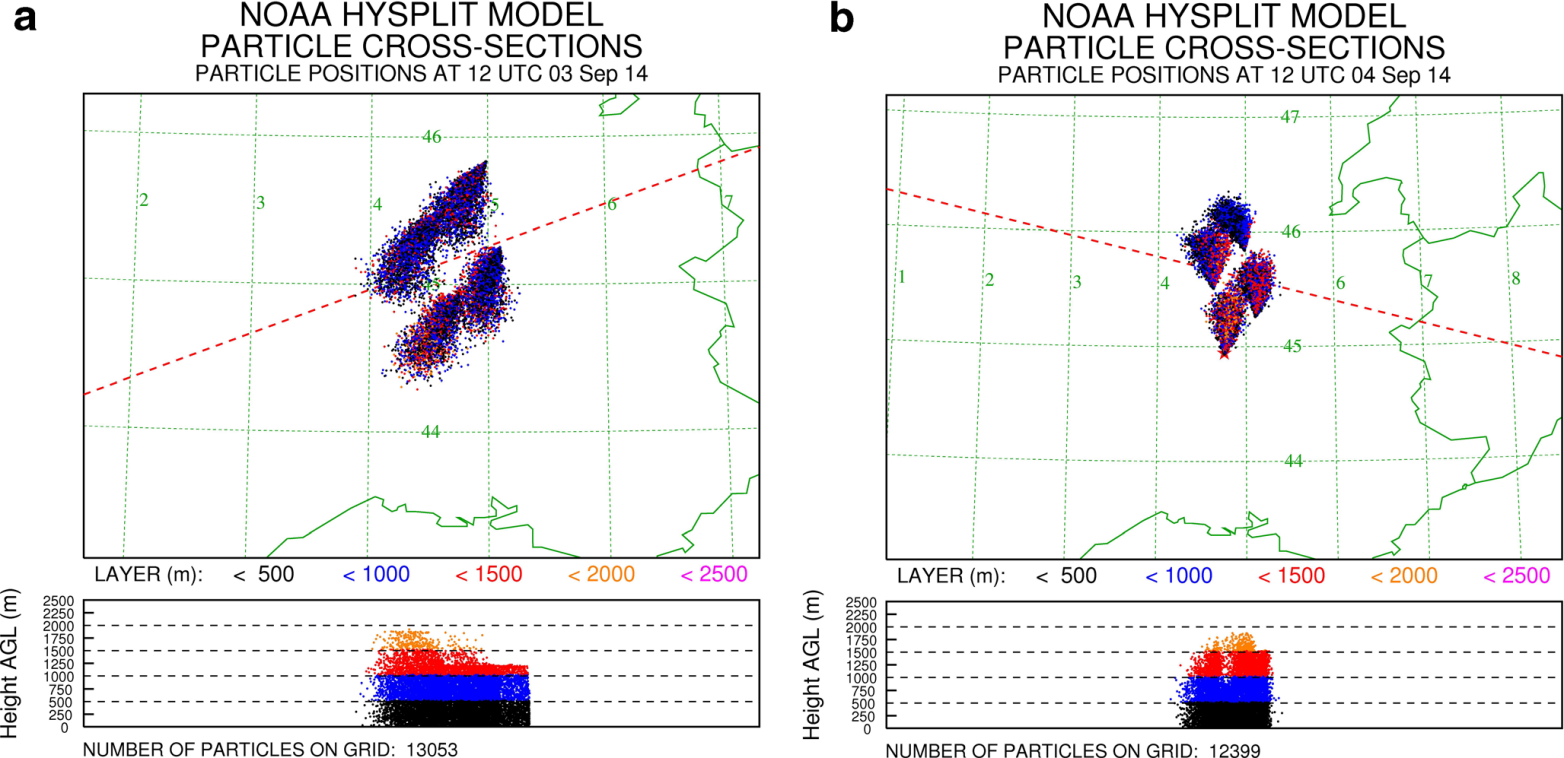
The output of the HYSPLIT model calculations of the distribution of particles released from 6 to 12 am in the Rhône Valley in France on 3 September 2014 (**a**) and 4 September 2014 (**b**)

The HYSPLIT_4 dispersion model was run again to determine whether the *Ambrosia* pollen produced in the most heavily infected areas in France (Thibaudon et al. 2014) could reach high altitudes. The particle cloud tended to go south on 3 September, but on 4 September, the particles reaching the higher levels went northward (Fig. 5). From particles remaining airborne after release, 2.5 % reached between 1471.8 and 2482.1 m, which is the altitudinal range of back trajectories arriving at Leiden when the pollen grains were recorded in the trap (Table 1). Calculations of particle concentration distribution carried out on 3–4 September 2014 confirmed that *Ambrosia* pollen grains could have reached sufficiently high above the ground to enter into the airstream moving towards Leiden (Fig. 5, Table 1).

The Rhône Valley has previously been identified as a potential source of *Ambrosia* pollen for Catalonia (Belmonte et al. 2000) and Switzerland (Taramarcaz et al. 2005), but this is the first time that it has been identified as a potential source of *Ambrosia* pollen in northwest Europe. The Rhône Valley is a known centre of *Ambrosia* in Europe and is closer to Leiden and Leicester than the Pannonian Plain; however, this study has shown that under these conditions, only a fraction of pollen released from France reached northwest Europe. In addition, the uncertainty resulting from orographically forced meteorology within the Rhône Valley cannot be resolved with default HYSPLIT_4 input data. Focused studies in such a region require much more detailed data, e.g. from the Weather Research and Forecast model as described by Hernandez-Ceballos et al. (2014). This suggests that the Pannonian Plain should still be considered to be the main source of the LTD *Ambrosia* pollen in Europe (Table 1).

**Fig. 6:**
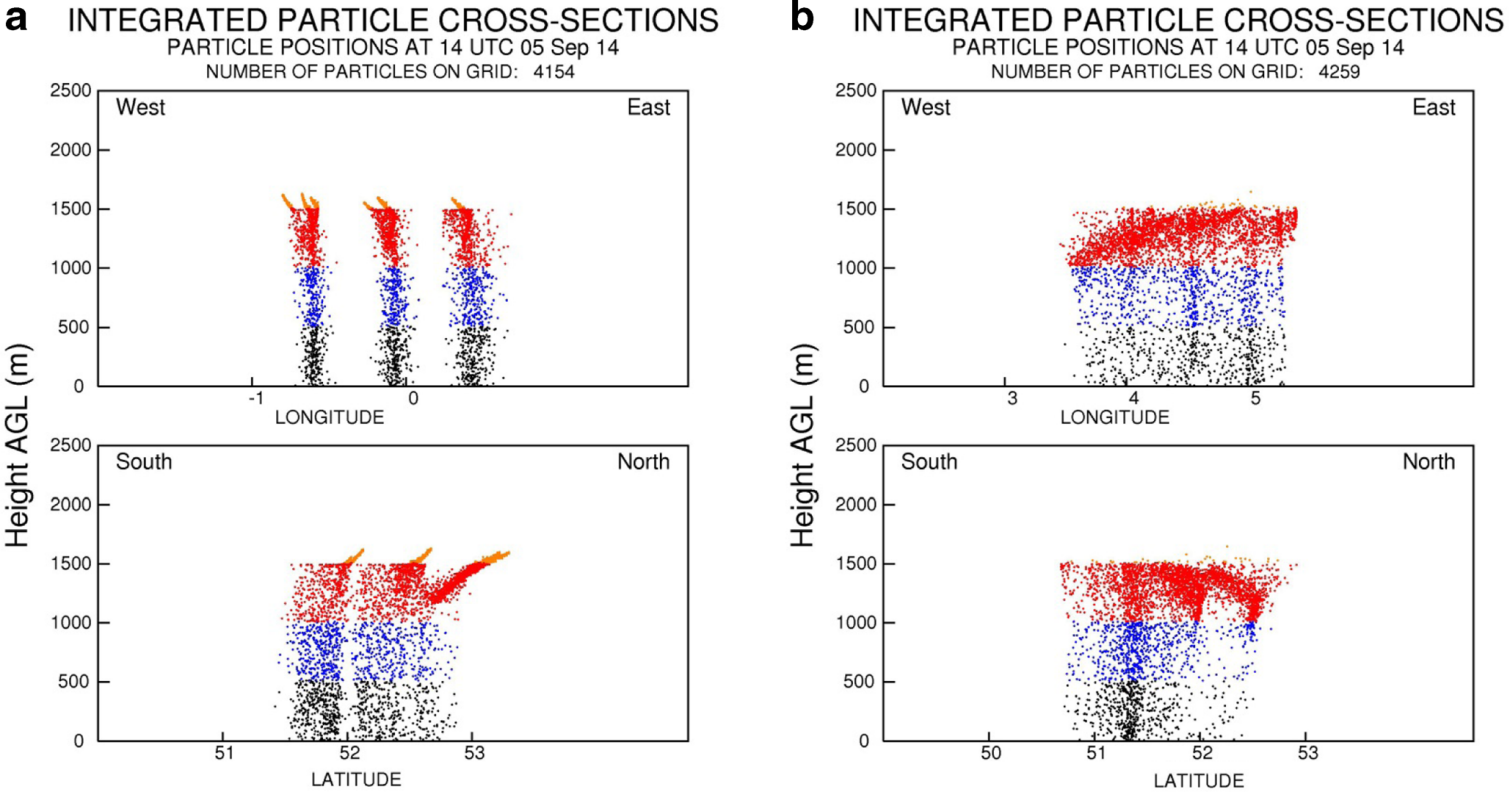
The output of the HYSPLIT model calculations of the distribution of particles released from 6 to 14 h at the location air masses pass on 5 September 2014, 6 h before arrival to Leicester (**a**) and Leiden (**b**)

HYSPLIT_4 simulations of particle deposition from the high altitude air masses, before they reached Leicester and Leiden, confirm that atmospheric conditions would have allowed for the deposition of airborne *Ambrosia* pollen to ground level in areas where surface pollen measurements took place (Fig. 6).

Several aspects of back trajectories are limited in respect to analysing air mass patterns. Earlier *Ambrosia* studies by Stach et al. (2007) and Šikoparija et al. (2009) used the Danish ACDEP model to calculate trajectories (Skjøth et al. 2002). This was a 2D trajectory model where the air masses followed the σ–level 0.925 wind vectors and 0.25° meteorological input. This approach (e.g. terrain following coordinates or isobaric coordinates) is conceptually simpler, but it neglects the vertical wind component (Stohl 1998), which means that errors in the calculation of 2D trajectories can accumulate faster with transport distance than for 3D trajectories. Current practice is therefore to use 3D trajectories, most commonly in relation to *Ambrosia* by using the HYSPLIT model (e.g. Makra et al. (2010); Saulienė et al. (2011); Zemmer et al. (2012) and recently Sommer et al. (2015)). Spatial and temporal resolution in the input data is, however, also very important as demonstrated by Skjøth et al. (2002) and Hernandez-Ceballos et al. (2014). These studies suggest that coastal effects and complex terrain often affect the meteorology on scales that are relevant for pollen transport and more detailed input to HYSPLIT or ACDEP provided substantially better output data, thus improving the analytical results. The effect on spatial and temporal resolution, however, depends on the atmospheric physics during the pollen episodes. Simulations of large scale flows will generally be less affected by increased resolution. Conversely, simulations of frontal zones, convective zones and orographic force flow will be heavily affected (e.g. Hernandez-Ceballos et al. 2014). In our case, there are generally large scale flows over the Pannonian Plain towards Leiden, while the flow in the Rhône Valley could be affected by complex terrain. As such, the findings relating to the Rhône Valley are uncertain due to limitations in resolving complex flows in this area.

It is not known whether such episodes of LDT have any consequences for the prevalence of sensitization to *Ambrosia* pollen. Threshold values required for *Ambrosia* pollen to induce symptoms differ among different studies, ranging from 1 to 3 P m^−3^ for “first symptoms to start” to 50 P m^−3^ for “60–80% of the sensitized patients to show symptoms” (de Weger et al. 2012; Déchamp et al. 1997). The public Internet platform in the Netherlands (Allergieradar.nl), where sufferers can enter their symptom scores (de Weger et al. 2014), did not show increases in numbers of entries or symptom severity during the studied period. Although it is important to mention that the number of entries was very low during that period. Furthermore, it is a matter of debate whether pollen that have been exposed to extreme circumstances during LDT have preserved its allergenic capacity (Cecchi et al. 2010).

## Conclusion

This study indicates that the *Ambrosia* pollen grains recorded at the beginning of September 2014 in Leicester and Leiden were probably not produced by local sources in response to range expansion due to climate change, but transported long distances from potential source regions in east Europe, i.e. the Pannonian Plain and Ukraine, as well as the Rhône Valley in France. As a result, this again confirms that *Ambrosia* pollen can be transported long distances from potential source regions, this time to the northwest fringes of Europe. In addition, we have shown that, using a dispersion model, *Ambrosia* pollen released from the Pannonian Plain reached high enough altitudes to enter westward-moving air masses and then settle out of the atmosphere to reach monitoring stations at ground level where they were recorded. This pollen released from the Pannonian Plain could augment the pollen moving west from more easterly areas such as Ukraine. The occurrence of an occluded front during the period helped to lift the pollen grains high into the atmosphere where they could be transported to northwest Europe. Furthermore, for the first time, we have identified the Rhône Valley in France as being a potential source of *Ambrosia* pollen in northwest Europe, albeit only a minor contributor compared to the Pannonian Plain. This study highlights the importance of the HYSPLIT dispersion model as a tool for distinguishing between LDT events and range expansion of an invasive, highly allergenic plant, an important distinction for plant and health management strategies.

## Electronic supplementary material

ESM 1 (DOCX 54 kb)
