# Supplementary material for: The long distance transport of airborne Ambrosia pollen to the UK and the Netherlands from Central and south Europe
Source: Int J Biometeorol. 2016 Apr 27;60(12):1829–39. doi: 10.1007/s00484-016-1170-7 (PMC5127884; doi:10.1007/s00484-016-1170-7)

**Supplementary Information: The long distance transport of airborne *Ambrosia* pollen to the UK and the Netherlands from Central and South Europe**

Letty A. de Weger^1*^, Catherine H. Pashley^2^, Branko Šikoparija^3, 4^, Carsten A. Skjøth^5^, Idalia Kasprzyk^6^, Łukasz Grewling^7^, Michel Thibaudon^8^, Donat Magyar^9^ and Matt Smith^7^

^1^Department of Pulmonology, Leiden University Medical Centre, Leiden, The Netherlands;

^2^Institute for Lung Health, Department of Infection, Immunity and Inflammation, University of Leicester, UK

^3^Laboratory for Palynology, Department of Biology and Ecology, University of Novi Sad Faculty of Sciences,

^4^BioSense Institute - Institute for research and development of information technology in biosystems, Novi Sad, Serbia

^5^National Pollen and Aerobiology Research Unit, Institute of Science and the Environment, University of Worcester, Henwick Grove, Worcester

^6^Departament of Environmental Biology, University of Rzeszów, Rzeszów, Poland

^7^Laboratory of Aeropalynology, Faculty of Biology, Adam Mickiewicz University, Poznań, Poland

^8^Reseau National de Surveillance Aerobiologique (RNSA), Brussieu, France;

^9^National Public Health Center, Department of Aerobiology and Air Hygiene, Budapest, Hungary

Corresponding author:Dr. Letty A. de Weger, Department of Pulmonology, Leiden University Medical Center, POBox 9600, 2300RC, Leiden, The Netherlands. Tel: +31-(0)71-5263578. Fax : +31-(0)71 5266927. e-mail: l.a.de_weger@lumc.nl

| Suppl. Table 1. Emission points chosen to asses the potential for particles to reach ground level monitoring stations at Leicester after traveling at ~1500m altitude. The exact location was determined as position where cluster of back-trajectories was situated six hours before arrival to Leicester on 5 September at 12:00 when 84 P m^-3^ was recorded. | | | |
| --- | --- | --- | --- |
| number | latitude | longitude | height |
| 1 | 52.961 | -1.079 | 1481.7 |
| 2 | 53.561 | -1.072 | 1595.4 |
| 3 | 52.362 | -1.157 | 1543.5 |
| 4 | 53.636 | -1.499 | 1580.1 |
| 5 | 53.006 | -1.554 | 1619.7 |
| 6 | 52.461 | -1.684 | 1610.8 |
| 7 | 53.554 | -0.588 | 1588.7 |
| 8 | 52.950 | -0.599 | 1448.1 |
| 9 | 52.329 | -0.645 | 1424.3 |

| Suppl. Table 2. Emission points chosen to asses possibility for particles to reach the ground level monitoring station at Leiden after traveling at ~1500m altitude. Exact location was determined as position where cluster of back-trajectories was situated six hours before arrival to Leiden on 5 September at 12:00 when 59 P m^-3^was recorded. | | | |
| --- | --- | --- | --- |
| number | latitude | longitude | height |
| 1 | 51.517 | 5.165 | 1436.6 |
| 2 | 51.960 | 5.325 | 1440.0 |
| 3 | 51.139 | 4.938 | 1472.9 |
| 4 | 51.724 | 5.843 | 1443.7 |
| 5 | 51.312 | 5.673 | 1407.9 |
| 6 | 50.870 | 5.372 | 1476.4 |
| 7 | 52.270 | 4.842 | 1435.9 |
| 8 | 51.821 | 4.673 | 1451.3 |
| 9 | 51.457 | 4.490 | 1456.1 |

Suppl. Table 3. Bihourly concentrations of airborne *Ambrosia* pollen recorded at monitoring sites in Northwestern Europe (Leicester and Leiden ), Eastern Europe (Poznan and Rzeszów), the Pannonian Plain(Nyíregyháza, Debrecen, Kecskemét and Sombor) and in the Rhône Valley (Lyon, and Rousillon).

|  | **station** | **Leicester** | **Leiden** | **Poznan** | **Rzeszów** | **Nyíregyháza** | **Debrecen** | **Kecskemét** | **Sombor** | **Lyon** | **Rousillon** |
| --- | --- | --- | --- | --- | --- | --- | --- | --- | --- | --- | --- |
|  | **Lat** | **52,38** | **52,17** | **52,42** | **50,07** | **47,95** | **47,54** | **46,90** | **45,77** | **45,75** | **45,22** |
|  | **Lon** | **-1.12** | **4,48** | **16,88** | **22,00** | **21,71** | **21,66** | **19,71** | **19,11** | **4,83** | **4,48** |
| **9-1-2014** | **00-02h** | **0** | **0** | **0** | **12** | **317** | **63** | **245** | **727** | **35** | **12** |
|  | **02-04h** | **0** | **0** | **0** | **18** | **139** | **63** | **467** | **317** | **12** | **140** |
|  | **04-06h** | **0** | **0** | **0** | **6** | **0** | **114** | **175** | **173** | **0** | **992** |
|  | **06-08h** | **0** | **0** | **0** | **0** | **25** | **127** | **1330** | **144** | **0** | **747** |
|  | **08-10h** | **0** | **0** | **0** | **0** | **2029** | **2422** | **2252** | **130** | **0** | **175** |
|  | **10-12h** | **0** | **0** | **0** | **12** | **4083** | **2092** | **338** | **1520** | **12** | **70** |
|  | **12-14h** | **0** | **0** | **0** | **0** | **3322** | **1928** | **805** | **2211** | **23** | **105** |
|  | **14-16h** | **0** | **0** | **0** | **0** | **2612** | **571** | **373** | **814** | **23** | **70** |
|  | **16-18h** | **0** | **0** | **0** | **0** | **888** | **254** | **140** | **943** | **35** | **35** |
|  | **18-20h** | **0** | **0** | **0** | **6** | **621** | **178** | **93** | **497** | **23** | **35** |
|  | **20-22h** | **0** | **0** | **0** | **6** | **254** | **304** | **47** | **101** | **0** | **23** |
|  | **22-24h** | **0** | **0** | **0** | **0** | **38** | **317** | **70** | **122** | **12** | **12** |
| **9-2-2014** | **00-02h** | **0** | **0** | **0** | **12** | **89** | **63** | **47** | **36** | **0** | **12** |
|  | **02-04h** | **0** | **0** | **0** | **49** | **38** | **13** | **70** | **72** | **0** | **35** |
|  | **04-06h** | **0** | **0** | **0** | **43** | **0** | **51** | **70** | **122** | **0** | **222** |
|  | **06-08h** | **0** | **0** | **0** | **0** | **228** | **292** | **1587** | **1440** | **0** | **350** |
|  | **08-10h** | **0** | **0** | **0** | **74** | **3082** | **3373** | **2053** | **1671** | **0** | **268** |
|  | **10-12h** | **0** | **0** | **0** | **377** | **4134** | **2917** | **770** | **1181** | **23** | **23** |
|  | **12-14h** | **0** | **0** | **0** | **377** | **3868** | **850** | **467** | **655** | **35** | **140** |
|  | **14-16h** | **0** | **0** | **0** | **247** | **1623** | **241** | **408** | **684** | **12** | **23** |
|  | **16-18h** | **0** | **0** | **0** | **198** | **850** | **850** | **397** | **756** | **12** | **12** |
|  | **18-20h** | **0** | **0** | **0** | **154** | **697** | **431** | **397** | **238** | **12** | **0** |
|  | **20-22h** | **0** | **0** | **5** | **198** | **317** | **216** | **233** | **158** | **12** | **35** |
|  | **22-24h** | **0** | **0** | **0** | **228** | **431** | **241** | **187** | **122** | **0** | **12** |
| **9-3-2014** | **00-02h** | **0** | **0** | **0** | **86** | **241** | **216** | **583** | **101** | **0** | **47** |
|  | **02-04h** | **0** | **0** | **0** | **80** | **469** | **596** | **968** | **36** | **0** | **152** |
|  | **04-06h** | **0** | **0** | **0** | **68** | **1332** | **1725** | **2007** | **137** | **0** | **1575** |
|  | **06-08h** | **0** | **0** | **0** | **68** | **2917** | **5111** | **3803** | **65** | **12** | **560** |
|  | **08-10h** | **0** | **0** | **0** | **86** | **3462** | **3995** | **1237** | **799** | **0** | **315** |
|  | **10-12h** | **0** | **0** | **0** | **62** | **1826** | **1978** | **805** | **2550** | **23** | **152** |
|  | **12-14h** | **0** | **0** | **0** | **154** | **1484** | **2232** | **1108** | **1909** | **12** | **82** |
|  | **14-16h** | **0** | **0** | **0** | **136** | **1699** | **1509** | **1178** | **338** | **23** | **35** |
|  | **16-18h** | **0** | **0** | **0** | **93** | **1103** | **1268** | **537** | **7** | **35** | **82** |
|  | **18-20h** | **0** | **0** | **0** | **68** | **406** | **482** | **245** | **29** | **0** | **35** |
|  | **20-22h** | **6** | **15** | **0** | **25** | **254** | **368** | **397** | **43** | **0** | **35** |
|  | **22-24h** | **0** | **7** | **0** | **0** | **304** | **406** | **560** | **36** | **0** | **23** |
| **9-4-2014** | **00-02h** | **0** | **0** | **0** | **6** | **482** | **190** | **1190** | **173** | **0** | **47** |
|  | **02-04h** | **0** | **7** | **0** | **6** | **571** | **609** | **1097** | **101** | **0** | **35** |
|  | **04-06h** | **0** | **52** | **0** | **12** | **533** | **1027** | **1925** | **194** | **0** | **23** |
|  | **06-08h** | **0** | **22** | **0** | **19** | **1737** | **3830** | **2030** | **526** | **0** | **1085** |
|  | **08-10h** | **0** | **22** | **0** | **37** | **3868** | **3652** | **933** | **1527** | **0** | **875** |
|  | **10-12h** | **6** | **59** | **0** | **43** | **3399** | **2866** | **548** | **1116** | **12** | **642** |
|  | **12-14h** | **6** | **45** | **0** | **68** | **2967** | **3132** | **467** | **1116** | **70** | **210** |
|  | **14-16h** | **26** | **22** | **0** | **62** | **2739** | **1775** | **607** | **511** | **210** | **93** |
|  | **16-18h** | **19** | **30** | **0** | **25** | **1966** | **875** | **863** | **792** | **268** | **105** |
|  | **18-20h** | **6** | **52** | **5** | **6** | **710** | **659** | **233** | **209** | **198** | **58** |
|  | **20-22h** | **52** | **45** | **0** | **12** | **659** | **241** | **233** | **130** | **117** | **23** |
|  | **22-24h** | **45** | **30** | **5** | **6** | **393** | **292** | **210** | **288** | **128** | **35** |
| **9-5-2014** | **00-02h** | **71** | **30** | **0** | **6** | **241** | **139** | **210** | **65** | **35** | **47** |
|  | **02-04h** | **26** | **52** | **0** | **0** | **63** | **165** | **245** | **14** | **23** | **58** |
|  | **04-06h** | **39** | **37** | **0** | **0** | **178** | **444** | **1202** | **65** | **47** | **117** |
|  | **06-08h** | **19** | **22** | **0** | **0** | **342** | **1230** | **2368** | **720** | **47** | **560** |
|  | **08-10h** | **58** | **37** | **11** | **25** | **3563** | **2080** | **852** | **1044** | **82** | **782** |
|  | **10-12h** | **58** | **0** | **58** | **6** | **3449** | **824** | **478** | **727** | **93** | **513** |
|  | **12-14h** | **84** | **59** | **53** | **37** | **2524** | **799** | **443** | **281** | **58** | **257** |
|  | **14-16h** | **13** | **45** | **5** | **43** | **2739** | **659** | **420** | **101** | **47** | **222** |
|  | **16-18h** | **45** | **37** | **16** | **43** | **2004** | **812** | **175** | **94** | **93** | **163** |
|  | **18-20h** | **0** | **37** | **5** | **12** | **1649** | **723** | **58** | **50** | **35** | **140** |
|  | **20-22h** | **6** | **15** | **11** | **25** | **2016** | **533** | **93** | **22** | **23** | **12** |
|  | **22-24h** | **0** | **15** | **0** | **12** | **1496** | **304** | **35** | **7** | **12** | **12** |

Suppl. Figure 1. Average daily *Ambrosia* pollen count in Leicester and Leiden from 1 September to 2 October 2014.


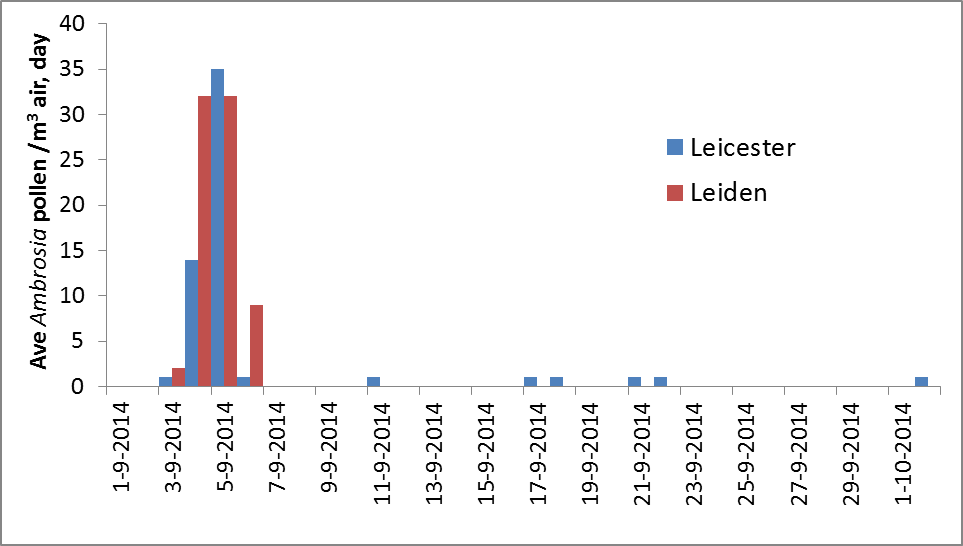

Supplement: Supplementary file 1 — (DOCX 54 kb) [file 484_2016_1170_MOESM1_ESM.docx]
